# Additive Manufacturing of PLA-Based Composites Using Fused Filament Fabrication: Effect of Graphene Nanoplatelet Reinforcement on Mechanical Properties, Dimensional Accuracy and Texture

**DOI:** 10.3390/polym11050799

**Published:** 2019-05-04

**Authors:** Miguel Ángel Caminero, Jesús Miguel Chacón, Eustaquio García-Plaza, Pedro José Núñez, José María Reverte, Jean Paul Becar

**Affiliations:** 1 Escuela Técnica Superior de Ingenieros Industriales, INEI, Universidad de Castilla-La Mancha, Campus Universitario s/n, 13071 Ciudad Real, Spain; eustaquio.garcia@uclm.es (E.G.-P.); pedro.nunez@uclm.es (P.J.N.); 2 Escuela Técnica Superior de Ingenieros Industriales, IMACI, Universidad de Castilla-La Mancha, Campus Universitario s/n, 13071 Ciudad Real, Spain; jesusmiguel.chacon@uclm.es (J.M.C.); josemaria.reverte@uclm.es (J.M.R.); 3 Laboratoire de Mathématiques et leurs Applications de Valenciennes, University Polytechnique Hauts-de-France, FR CNRS 2956, F-59313 Valenciennes, France; jean-paul.becar@uphf.fr

**Keywords:** 3D printing, fused filament fabrication, graphene nanoplatelets (GNPs), polylactic acid (PLA) composites, mechanical characterization, dimensional accuracy, surface texture

## Abstract

Fused filament fabrication (FFF) is a promising additive manufacturing (AM) technology due to its ability to build thermoplastics parts with advantages in the design and optimization of models with complex geometries, great design flexibility, recyclability and low material waste. This technique has been extensively used for the manufacturing of conceptual prototypes rather than functional components due to the limited mechanical properties of pure thermoplastics parts. In order to improve the mechanical performance of 3D printed parts based on polymeric materials, reinforcements including nanoparticles, short or continuous fibers and other additives have been adopted. The addition of graphene nanoplatelets (GNPs) to plastic and polymers is currently under investigation as a promising method to improve their working conditions due to the good mechanical, electrical and thermal performance exhibited by graphene. Although research shows particularly promising improvement in thermal and electrical conductivities of graphene-based nanocomposites, the aim of this study is to evaluate the effect of graphene nanoplatelet reinforcement on the mechanical properties, dimensional accuracy and surface texture of 3D printed polylactic acid (PLA) structures manufactured by a desktop 3D printer. The effect of build orientation was also analyzed. Scanning Electron Microscope (SEM) images of failure samples were evaluated to determine the effects of process parameters on failure modes. It was observed that PLA-Graphene composite samples showed, in general terms, the best performance in terms of tensile and flexural stress, particularly in the case of upright orientation (about 1.5 and 1.7 times higher than PLA and PLA 3D850 samples, respectively). In addition, PLA-Graphene composite samples showed the highest interlaminar shear strength (about 1.2 times higher than PLA and PLA 3D850 samples). However, the addition of GNPs tended to reduce the impact strength of the PLA-Graphene composite samples (PLA and PLA 3D850 samples exhibited an impact strength about 1.2–1.3 times higher than PLA-Graphene composites). Furthermore, the addition of graphene nanoplatelets did not affect, in general terms, the dimensional accuracy of the PLA-Graphene composite specimens. In addition, PLA-Graphene composite samples showed, in overall terms, the best performance in terms of surface texture, particularly when parts were printed in flat and on-edge orientations. The promising results in the present study prove the feasibility of 3D printed PLA-graphene composites for potential use in different applications such as biomedical engineering.

## 1. Introduction

Additive manufacturing (AM) is one of the most promising areas in the manufacturing of components from prototypes to functional structures with complex geometries and is revolutionizing different important industrial areas such as in aerospace, automotive, semiconductor or biomedical applications [1,2,3,4,5,6,7,8,9]. Additive manufacturing is distinguished from traditional manufacturing techniques, such as casting and machining, by its ability to handle complex shapes with great flexibility and without the typical waste [7,8,10,11]. Among the different AM techniques, 3D printing based on fused filament fabrication (FFF)—using thermoplastic polymers that require low melting temperature and rapid solidification times—is widely adopted for the simplicity of the method and its relatively low cost and low material wastage [3,8,11,12,13]. FFF forms a 3D geometry through the deposition of successive layers of extruded thermoplastic filament, such as acrylonitrile butadiene styrene (ABS), polylactic acid (PLA), polypropylene (PP) or polyethylene (PE). In addition, engineering thermoplastics with improved mechanical performance, such as polyamide (Nylon), polycarbonate (PC), polyetheretherketone (PEEK), polyetherimide (PEI), polyethersulfone (PES) or polyphenylene sulfide (PPS) is also possible [14]. However, eco-friendly polymeric materials with good physical properties are of major concern for FFF. For example, PLA has great worldwide demand due to versatile applicability in packaging, pharmaceuticals, textiles, automotive, and biomedical and tissue engineering [15,16,17,18]. It has been widely investigated for biomedical applications due to its biodegradability, bioresorbability and biocompatibility [15].

The interest in FFF abilities has expanded to include functional finished parts in addition to rapid prototyping. Moreover, the cost of manufacturing small series or unique parts can be significantly reduced [19]. This has motivated research into the mechanical, electrical, thermal and other properties characterizations and improvements of parts manufactured employing this technology [8]. Despite the apparent advantage over more traditional methods, FFF printed parts often suffer from poor mechanical characteristics, limiting their broader adaption for end-use, fully functional and load bearing components [20,21,22,23,24]. Furthermore, mechanical properties of parts manufactured by conventional FFF 3D printing are inherently poor because of the thermoplastic resin used, although the optimization of processing parameters, such as build orientation, layer thickness or feed rate, has been investigated for improving the mechanical properties of thermoplastic parts in a limited number of studies [9,13,25,26]. However, regardless of parameter optimization, FFF printed parts still exhibit lower properties compared to those obtained by conventional polymer processing methods such as compression or injection molding [3]. Additionally, the quality of final fully-formed FFF parts in terms of dimensional accuracy or surface roughness is affected by part intricacy, the corresponding print path, and the differential cool-down and solidification of the individual rasters, among other factors [27,28,29]. Such drawbacks restrict the wide industrial application of 3D printed thermoplastic polymers, leaving prototyping as the primary application [20]. Hence, it is very necessary to understand the shortcomings of the FFF process for its better application in modern industries.

3D printing of polymer composites with enhanced mechanical properties solves the previous limitations by combining the matrix and reinforcements to achieve a system with more useful structural or functional properties non-attainable by any of the constituent alone [8,30]. Incorporation of particles, fibers or nanomaterial reinforcements into polymers permits the fabrication of polymer matrix composites that are characterized by high performance and excellent functionality [7,8,31]. Various reinforcement, such as short fibers, including chopped carbon or glass fibers, have been used in a limited number of studies with a moderate improvement of mechanical properties [10,24,32,33,34,35,36,37]. In most studies, short fibers were embedded in ABS or Nylon thermoplastic filaments, prior to being loaded into the printer. The possibility of employing continuous fiber reinforced thermoplastic composites may lead to products with much higher mechanical performance, which are potentially useful for advanced applications [38,39]. However, their processing is not commonplace, and a specially designed printer is required [3,20,22,30,40].

The allure of the recent introduction of nanotechnology into this innovative field is due to the remarkable improvements and diversifications in properties of the resulting 3D printed materials, exhibiting optimized properties and multifunctionality. In particular, there is increasing interest in the development of high-performance composites suitable for 3D printing, achieved via the introduction of nanomaterials with unique properties such as nanotubes and graphene and its derivatives in the polymer matrix. Graphene’s excellent mechanical, electrical and thermal properties make it an attractive candidate for the reinforcement of several polymers [41]. Graphene’s addition to polymer matrices has resulted in composites exhibiting superior mechanical strength while retaining its flexibility, as well as tailorable thermal and electrical conductivity because of the graphene network in the matrix [15,16,17,18]. However, PLA-graphene composite blends are currently being used for the fabrication of 3D-printed scaffolds for tissue engineering [16]. Although biocompatibility of graphene-reinforced PLA has been proven in previous studies, its potential application in load-bearing structures and the resultant performance under different loading conditions need to be evaluated [18,42]. Recently, a few studies have reported the successful development of graphene-based reinforced polymer composites for 3D printing [43,44,45]. However, in composites, the main challenge is to understand how to transfer the properties of graphene from the nanoscale to the macroscale. Although research shows particularly promising improvement in thermal and electrical conductivities of graphene-based nanocomposites [44,45,46,47], the primary goal of this research is to explore the initial steps toward improving the mechanical performance of 3D printed PLA-based nanocomposites that include graphene nanoplatelets (GNPs). The addition of graphene nanoplatelets to polymers is under investigation as a promising method to improve the mechanical and thermal properties of these materials. However, previous findings showed that the addition of other types of particles to PLA-based composites caused a decrease in the mechanical properties of the polymer composite used in 3D printing [48]. In addition, among the existing literature on mechanical properties of 3D printed PLA composites, there is a lack of study on their interlaminar bonding performance [37,38,39,49]. The interface bounding quality between layers and wires significantly influences the microstructure and mechanical properties of the resulting parts [39]. Moreover, further research is required to determine the quality of 3D printed composites parts as a function of different process parameters (build orientation, layer thickness or type of reinforcement) in terms of dimensional accuracy or surface roughness since the literature on the dimensional and surface texture characterization of 3D printed parts processed by the FFF technique is somewhat scarce [27].

In this study, commercially available polylactic acid (PLA), an enhanced PLA-based polymer (PLA 3D850) and graphene nanoplatelet reinforced PLA composite (PLA-Graphene) filaments were used to manufacture different samples by the FFF technique using a low-cost desktop 3D printer. The mechanical properties, in terms of tensile and three-point bending performance, are evaluated. In addition, the interlaminar bonding and impact performance of the 3D printed samples are also studied. The effect of the graphene reinforcement and build orientation are analyzed. A comparison of the mechanical performance, dimensional accuracy and surface texture between virgin PLA and reinforced PLA samples is also conducted. Finally, SEM images of failure samples are evaluated to determine the effects of the process parameters on failure modes.

The rest of the paper is organized as follows. First, the experimental methodology carried out in this study is briefly summarized with particular emphasis on specimen preparation, the 3D printing process and the experimental set-up. Thereafter, the key results of the investigation are summarized, and the effects of the different process parameters on the mechanical performance, dimensional accuracy and texture are highlighted. Finally, conclusions and extensions of this work are outlined.

## 2. Materials and Methods

### 2.1. Materials, 3D Printer and Specimen Preparation

**Figure 1:**
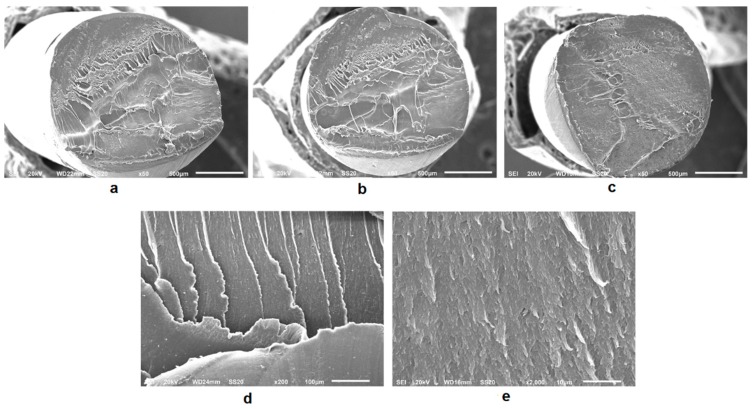
Cross-sectional images for printing wires (×50): (**a**) Polylactic acid (PLA); (**b**) PLA 3D850 and (**c**) PLA-Graphene filaments; (**d**,**e**) Enlarged PLA-Graphene views (200× and 2000×, respectively).

The goal of this study is to analyze the mechanical performance, dimensional accuracy and texture of PLA-graphene composite samples. Three different commercially available PLA-based filaments, with a diameter of 1.75 mm, have been analyzed: SMARTFIL^®^ PLA natural [50], a modified PLA-based polymer, SMARTFIL^®^ PLA 3D850 natural [51], both filaments manufactured by Smart Materials 3D (Jaén, Spain) and HDPlas^®^ PLA (PLA-graphene) manufactured by Haydale Ltd. (Carmarthenshire, UK) [52]. PLA 3D850 provides less thermal contraction and better mechanical properties than traditional PLA, making it ideal for high accuracy, high resolution and high-performance applications. In addition, PLA-graphene composite filament includes HDPlas^®^ functionalized graphene nanoplatelets (GNPs) of a planar size between 0.3–5 μm in order to improve dispersion and bonding within the PLA polymer. It is expected to improve thermal stability, print quality, the first layer and *z*-axis adhesion. Figure 1 shows the cross-sectional SEM images of the three wires. Samples were coated with a thin layer of gold to make them conductive. This required the use of a sputter-coater. It can be seen that there are some pores in the PLA-graphene wire, but no obvious pores in the unreinforced PLA-based wires. In addition, PLA-graphene wire depicts the presence of uniformly dispersed GNPs embedded in the polymeric PLA matrix.

The basic mechanical properties of PLA-based materials used in this work and typical ranges of mechanical properties for PLA and ABS materials manufactured by FFF technology provided by the manufacturers are presented in Table 1 for comparative purposes [12,13,50].

**Figure 2:**
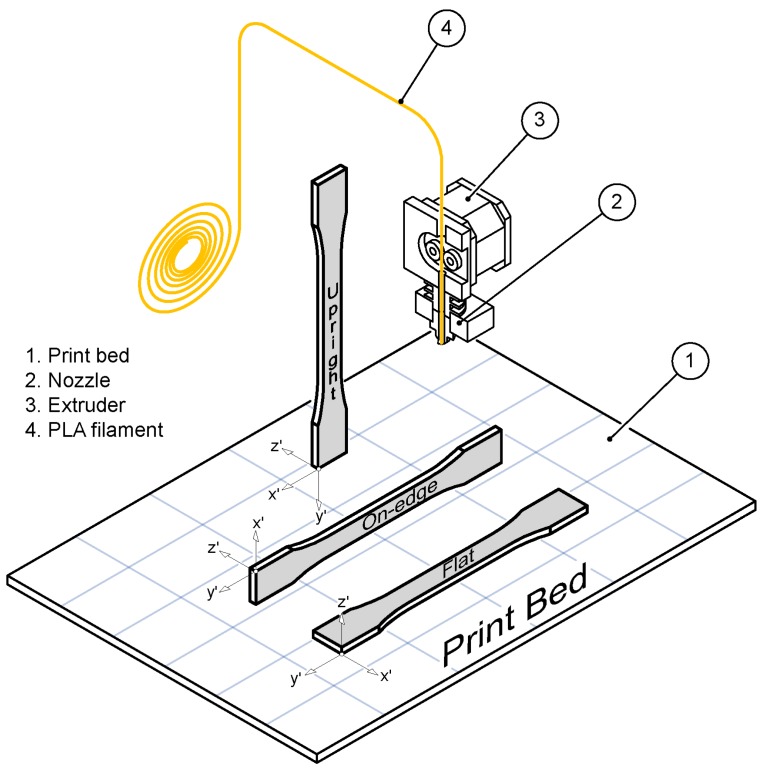
Witbox desktop 3D printer system. Details of the printing process and build orientation.

PLA-based samples were manufactured using a WitBox desktop 3D printer developed by BQ [53]. WitBox is a low-cost desktop printer that uses PLA-based filaments with a 0.4 mm nozzle size (Figure 2). WitBox can be controlled with any open source software. In this study, Cura software [54] was used to generate G-code files and to command and control all the process parameters.

**Figure 3:**
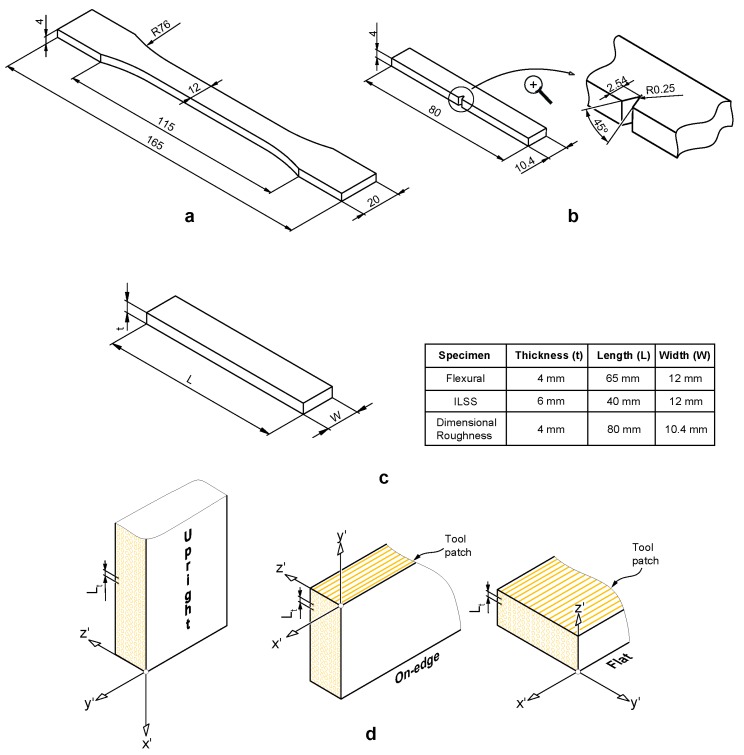
Standard specimens for mechanical testing. (**a**) Tensile specimen; (**b**) Charpy impact specimen; (**c**) Flexural, interlaminar shear strength (ILSS) and dimensional specimens; (**d**) Details of layer thickness (L_t_) and build orientations (Upright, On-edge and Flat) used in this study. Dimension are in mm.

There are no standard test methods for the mechanical characterization of parts manufactured using FFF. In this study, the recommendations of the ASTM standards D638 [55], D790 [56], D6110-10 [57] and D2344 [58] were followed for testing tensile, flexural, Charpy impact and interlaminar shear strength (ILSS) specimens, respectively. The geometry of the 3D printed specimens was modelled using SolidWorks software, exported as an STL file and imported to the 3D printing software. The main dimensions of the samples are shown in Figure 3.

### 2.2. Process Parameters

The mechanical, dimensional and surface finish properties of parts fabricated using FFF technology depend on the selection of process parameters [8,13]. In this study, three different build orientations were assessed (Figure 3d): Flat (F), On-edge (O)—where the fused filament deposition is positioned in the same direction as the tensile pull direction—and Upright (U)—in which layers were deposited perpendicular to the tensile pull direction. In order to focus on the mechanical performance of the three PLA-based materials, FFF process parameters such as layer thickness, feed rate, air gap, raster angle or temperature were fixed for all the samples. Table 2 shows the values of these parameters.

There is a broad spectrum of infill patterns, making it difficult to analyze the influence of raster patterns. In this study, solid samples filled with a perimeter raster were analyzed, which is where shell thickness was selected long enough to fill the sample with a raster angle of 0° (Figure 3d). In other words, the tool paths are the offsets from the perimeter with a distance equivalent to the nozzle size.

Each sample set consisted of five specimens for each orientation and material, with a total of 150 specimens for mechanical testing (tensile, 3-point bending, Charpy impact and ILSS specimens) and 45 specimens for dimensional and surface roughness characterization. Average values of the mechanical, dimensional and surface roughness tests were taken as the results. The manufactured specimens were stored in a dry box in order to minimize moisture absorption, which adversely affects the mechanical performance of PLA. Since the physical properties of many materials (especially thermoplastics) can vary depending on ambient temperature, tests were carried out according to the standards for room temperature.

### 2.3. Experimental Set-Up

A 50 kN universal electro-mechanical testing machine with a 5 kN load cell was used for the uniaxial tensile, 3-point bending and ILSS tests at a fixed loading rate of 2 mm/min, for both the tensile and 3-point bending tests, and 1 mm/min for short beam shear test (SBS) to obtain the ILSS strength. The selection of this displacement rate was in agreement with the displacement rate used in other studies [1,5,6,13,39] and was within the proposed ASTM test speed range of 1–5 mm/min. Tensile strain was measured using an MTS 634.14 high-performance axial extensometer. Charpy impact tests were performed to study the energy absorption of the different materials and orientations. To determine the impact damage strength of the 3D printed samples, a BOT 633 D Charpy test ring with a maximum energy of 10 J was used. The experimental data were processed following the corresponding standard. For more details of the different experimental set-up, the reader is referred to previous works [13,38,39].

For dimensional inspection, the optical measurement used a vision system Tesa-Visio 200 with a motorized zoom and CCD color camera. The axis ranges are X-200 mm, Y-100 mm, Z-150 mm, with a resolution of 0.001 mm; repeatability is defined by Ex, Ey *=* 2.0 + 10*L*/1000 µm, where *L* is the machine axis length in mm. In addition, surface texture was measured using a 3D Surface Profiling System Talysurf CLI 1000 with a range of 100 mm in the three axes (X, Y, Z). The Talysurf CLI 1000 system uses an inductive contact gauge with a 2.5 mm Z-axis range and a resolution of 40 nm. In addition, surface texture was measured by sampling a 10 x10 mm^2^ area in a flat position on the surface center point of the workpiece upper face, taking 334 profiles separated 24 µm. The surface roughness parameters analyzed were the *Arithmetic Mean Height* (Sa) and the *Maximum height* (Sz) [59] and for flatness deviation the *Root Mean Square Flatness Deviation* (Fltq) with *Least Squares Reference Plane* (LSPL) [60].

## 3. Results and Discussion

**Figure 4:**
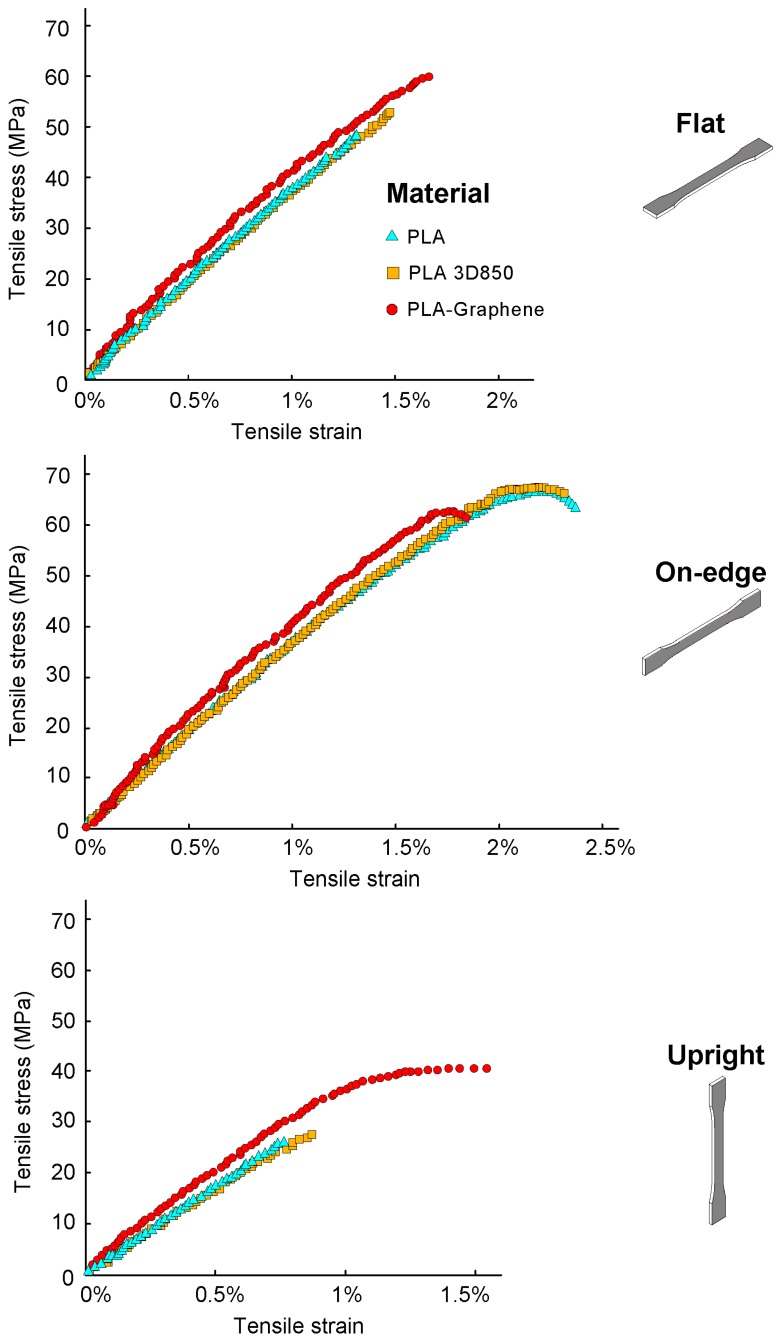
Average tensile stress–strain curves for the 3D printed PLA-based samples as a function of the material and the build orientation.

**Figure 5:**
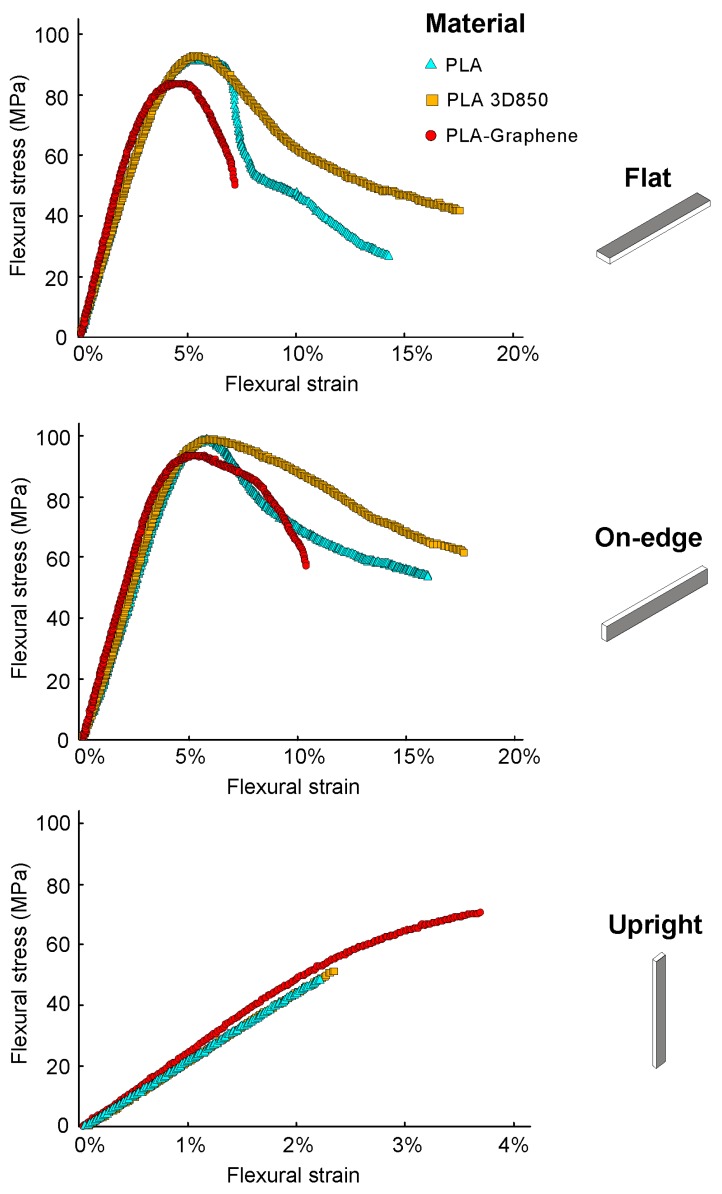
Average flexural stress–strain curves for the 3D printed PLA-based samples as a function of the material and the build orientation.

**Figure 6:**
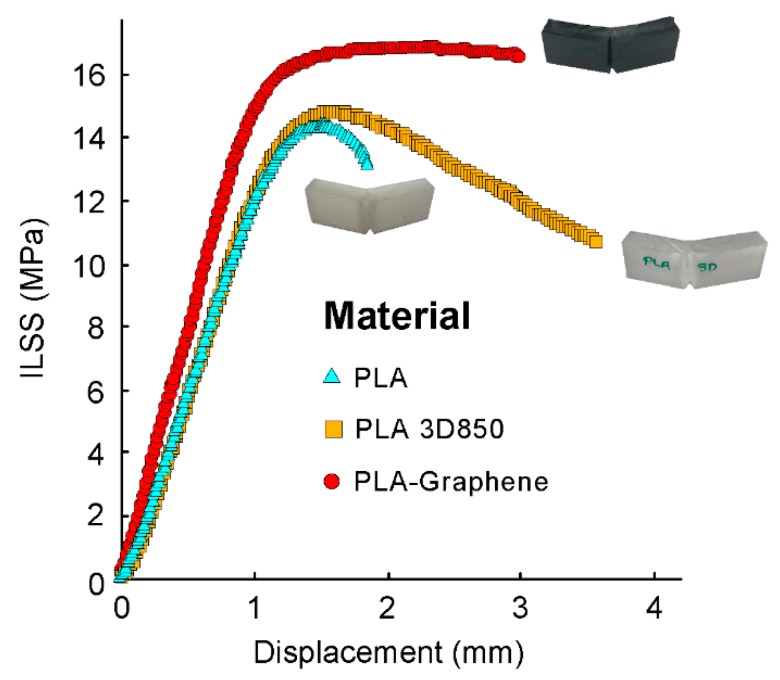
Average ILSS-displacement curves under different printing conditions. Cross-sectional optical micrographs showing the details of the failure modes for the different configurations.

Average and standard deviation of the test results of the maximum tensile and flexural strength (σ_t_, σ_f_) and stiffness (E_t_, E_f_) of the 3D printed PLA-based samples are tabulated in Table 3. In addition, average and standard deviation of the Charpy impact strength (E_C_) and the maximum interlaminar shear strength (τ_ILSS_) are tabulated in Table 4 and Table 5, respectively. Finally, Figure 4 and Figure 5 report some representative stress–strain curves for the tensile and flexural PLA-based samples with different process parameters in order to characterize and assess the different types of damage observed. Figure 6 reports some representative interlaminar shear strength–displacement curves and failure modes for the PLA-based samples.

The main effects of the build orientation and graphene reinforcement on the mechanical performance and dimensional accuracy and texture of 3D printed PLA-based samples are summarized in the following sections.

### 3.1. Effect of Build Orientation and Graphene Reinforcement on the Mechanical Performance of PLA-Based Samples

#### 3.1.1. Tensile and Flexural Performance of PLA-Based Samples

A first glance at the results of Table 3 and Figure 4 and Figure 5 reveals that the PLA-based samples exhibited a remarkable anisotropy. Build orientation significantly affected the mechanical properties. On-edge and flat orientations showed the highest values for maximum tensile and flexural strengths and stiffness, while Upright orientation resulted in the lowest ones. For example, On-edge PLA samples depicted an averaged increase in tensile strength of 154% compared with Upright ones. In the case of flexural performance, a further increase in average flexural strength between On-edge and Upright orientations was observed, increasing by 133%. More specifically, On-edge orientation depicted the highest value for the maximum tensile and flexural strength, except in the case of the PLA-Graphene composite. These results have confirmed the observations of previous studies [17,18]. These differences can be explained by considering two main failure modes: inter-layer fusion bond failure (inter-layer failure) and trans-layer failure. For the Upright orientation, the samples were pulled parallel to the layer deposition direction and the load was perpendicular to their fibers, resulting in inter-layer fusion bond failure. In this case, layer or fiber-to-fiber adhesion significantly affected tensile strength given that inter-layer fusion bonds between adjacent layers or fibers withstood most of the applied load and not the fibers themselves. A lower tensile strength than the individual fibers was expected [4,6,13,61,62,63].

In the cases of the On-edge and Flat orientations, the specimens were pulled perpendicular to the layer deposition direction and hence fibers were pulled parallel to the loading direction, resulting in trans-layer failure. In this case, individual fibers withstood most of the applied load and fiber breakage was observed [6,13,61,62,63]. If Flat and On-edge orientations were pulled parallel to the layer deposition direction, inter-layer failure is expected in a similar way to the Upright orientation with lower tensile strength than in the case of trans-layer failure.

In general, the results highlighted a brittle behavior for the Upright orientation. However, On-edge and Flat orientations showed a more ductile behavior, with higher plastic deformation. More specifically, On-edge samples depicted the value of maximum tensile deformation at fracture (Figure 4), with similar values for elastic modulus as in Flat samples, since more layers were pulled longitudinally. From a flexural point of view, Figure 5 shows that the trends of the flexural stress–strain behavior results were similar to the tensile ones, where Upright orientation depicted a brittle performance, and On-edge and Flat orientations showed a ductile behavior and plastic deformation with a similar amount of flexural deformation. These findings underscored that the selection of build orientation of the PLA-based samples had a crucial impact on the strength, stiffness and deformation at fracture.

In addition, the results of Figure 4 and Figure 5 show that there was no significant difference between PLA and PLA3D850 in terms of tensile and flexural behavior. The differences in mechanical performance were lower than 7%. However, it is shown that PLA-Graphene samples exhibited a slightly larger stiffness, E_t_, for each of the three orientations. GNPs offered higher stiffness with respect to PLA matrix, preventing the shear strain. This increase in the stiffness, E_t,_ suggest that there is a higher resistance to plastic deformation in the reinforced samples. This behavior can be attributed to the effective transfer of stress to the graphene reinforcement. The results are in accordance with previous works [15]. Furthermore, PLA-Graphene composite samples showed the best performance in terms of tensile and flexural stress and stiffness, except in the case of On-edge orientation. More specifically, PLA-Graphene composite showed a significant improvement of the tensile behavior over the other two materials in the case of Upright orientation. Upright PLA-Graphene samples depicted an averaged increase in tensile strength of 50.6% and 41.3% compared with Upright PLA and PLA 3D850 samples, respectively. In the case of flexural performance, a further increase in average flexural strength between Upright PLA-Graphene and PLA-based samples was observed, increasing by 68.1% and 49.4% compared with PLA and PLA 3D850, respectively. These results were in good agreement with previous findings [13,64]. The improvement in the mechanical properties of 3D printed PLA-Graphene composites indicates an enhanced interlayer adhesion and the refinement of 3D printing processing parameters could result in further improvement of the overall mechanical properties.

#### 3.1.2. Charpy Impact Performance of PLA-Based Samples

Table 4 depicts the average and standard deviation of the maximum impact strength (E_c_) as a function of the PLA material and build orientation. It can be observed that On-edge and Flat orientations showed higher values of impact strength than upright orientation. In the case of Flat samples, impact loading was parallel to the adjacent layers and they withstood most of the applied load. On the other hand, in the case of On-edge samples, impact loading was perpendicular to the individual layers, and they withstood most of the applied load. In addition, Flat samples exhibited the highest value of impact strength for the three PLA-based materials. These results were in line with those reported in references [38,65] for unreinforced thermoplastic materials using a desktop and an industrial grades material extrusion 3D printer [25].

Comparing impact strength, *E*_c_, of the PLA-Graphene specimens with PLA and PLA 3D850 specimens, it is observed that the impact strength, *E*_c_, of PLA and PLA 3D850 samples was about 1.2–1.3 times higher than the maximum impact strength of PLA-Graphene composite samples. Hence, the addition of GNPs tended to reduce the impact strength of the PLA composite samples. This can be explained by the brittle behavior exhibited by the PLA-Graphene composite samples in On-edge and Flat orientations. On the other hand, in the case of Upright oriented samples, PLA-Graphene composite samples showed similar or even higher ductility and mechanical performance compared to PLA and PLA 3D850 samples. This trend indicates an enhanced interlayer adhesion and it is in agreement with the findings of the previous section.

#### 3.1.3. Interlaminar Shear Strength Performance of PLA-Based Samples

The bonding performance of the different layers is a main factor for the mechanical strength of 3D printed thermoplastic parts. Figure 6 reports some representative interlaminar shear strength-displacement curves and failure modes for the ILSS samples with different materials in order to characterise and assess the different types of damage observed.

In addition, Table 5 shows average values and standard deviation of maximum interlaminar shear strength for the three PLA-based materials. The results revealed that PLA and PLA 3D 850 samples exhibited similar interlaminar shear strength. In addition, PLA 3D850 samples exhibited the maximum ductility in terms of maximum displacement, which is in good agreement with the trends observed for on-edge and flat orientations in flexural performance. On the other hand, PLA-Graphene composite samples showed the highest interlaminar shear strength. These results were in accordance with the enhanced interlaminar adhesion and performance showed by the PLA-Graphene samples under tensile and flexural loading.

**Figure 7:**
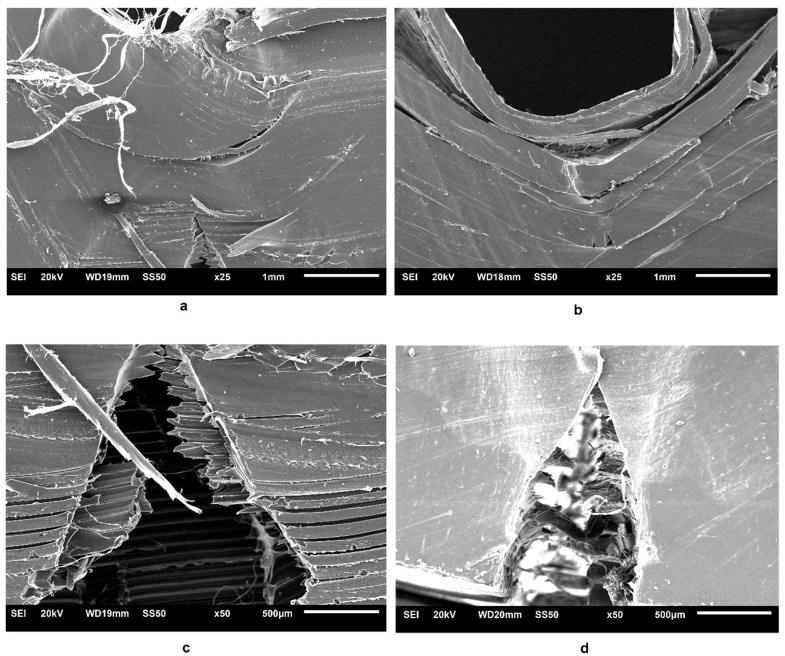
Scanning Electron Microscope (SEM) images showing details of the fractured surfaces of the ILSS samples. (**a**) Failure mode of PLA 3D850 samples (25×) and (**b**) failure mode of PLA-Graphene composite samples (25×) (**c**) and (**d**) Details of the fractured surfaces of PLA 3D 850 and PLA-Graphene samples, respectively (50×).

Finally, SEM examination of the cross-sectional ILSS samples was performed to obtain information on the interlayer performance. Figure 7 depicts SEM images showing details of the interlaminar shear failure surfaces of PLA 3D850 and PLA-Graphene samples. In general, the results highlighted interlaminar shear failure due to delamination of the thermoplastic layers in both materials (Figure 7a,b) and the initiation of breakage in the lower layers (Figure 7c,d). These results have confirmed the observations of previous studies [20,22,39,49].

### 3.2. Effect of Build Orientation and Graphene Reinforcement on the Dimensional Accuracy and Surface Roughness of PLA-Based Specimens

**Figure 8:**
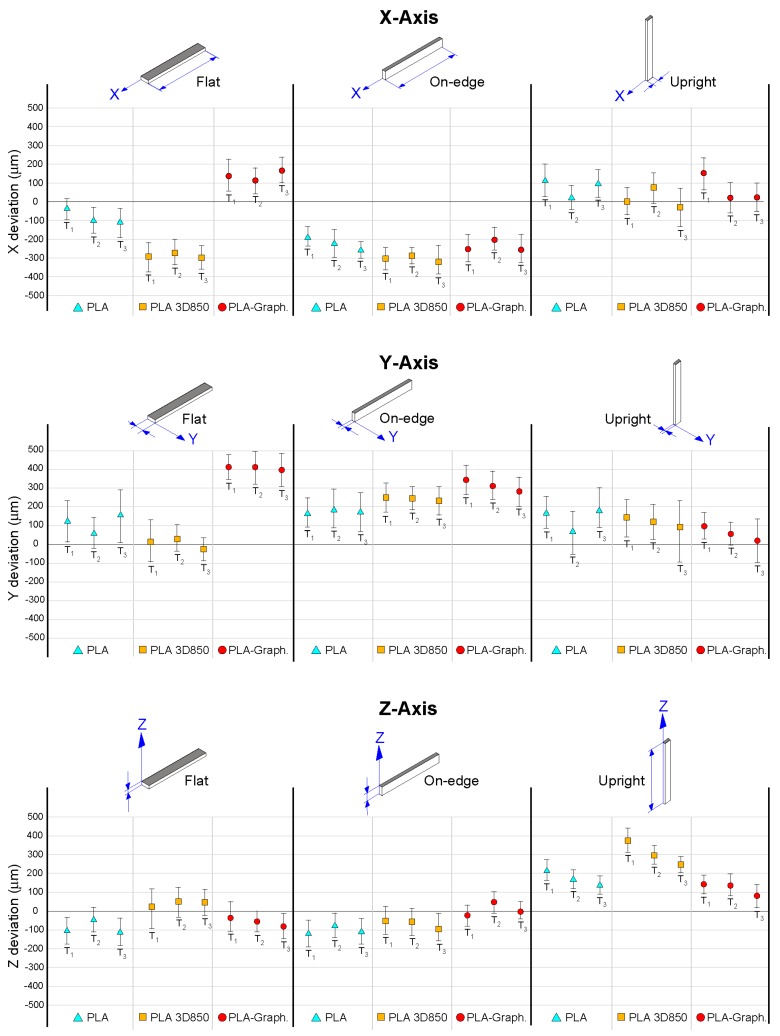
Dimensional accuracy results of PLA-based samples as a function of build orientation and the type of material. The results of three samples of each set are shown, including the mean, maximum and minimum values of each sample for comparative purposes.

**Figure 9:**
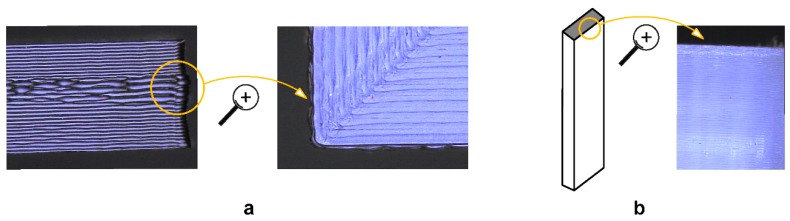
(**a**) Details of deformed layers and filaments for flat orientation. (**b**) Crystallized areas in upright orientation.

Table 6 and Figure 8 depict the dimensional accuracy of PLA-based samples measured in *X*-axis, *Y*-axis and *Z*-axis, respectively, showing the mean, the maximum and the minimum values of the three materials and build orientations analyzed in this study. The results of three samples of each set are shown. *X*-axis and *Y*-axis are related to the dimensional accuracy of deposited filaments in the same layer. On the other hand, *Z*-axis is associated with the dimensional accuracy of deposited layers. The results showed that the greatest differences in dimensional accuracy are linked with the build orientation. In the case of the *X*-axis, Flat and On-edge orientations depicted higher dimensional deviation than Upright orientation. More specifically, flat and on-edge PLA 3D samples showed a mean deviation of 288 ± 13 µm and 304 ± 16 µm, respectively, while upright PLA samples depicted a mean deviation of 78 ± 48 µm. The reason was that Upright orientation was affected by layer accumulation and a lower dimensional deviation was expected. In the case of the *Y*-axis, it depicted the highest mean dimensional deviation of all test with a maximum mean deviation of 407 ± 9 µm for Flat PLA-Graphene samples. These specimens showed defects in the middle layers of flat-oriented samples (Figure 9a), that resulted in a dimensional thickening in the X-Y plane. Finally, in the case of *Z*-axis, an improvement in the dimensional accuracy was observed with a maximum mean deviation of 100 µm for the three PLA-based materials—except in the case of upright orientation, due to a higher number of deposited layers. The reason was the development of crystallized areas in the deposited layers (Figure 9b), which increased the dimensional deviation. In short, although PLA samples showed the best dimensional performance with good repeatability, the addition of graphene nanoplatelets did not affect, in general terms, the dimensional accuracy of the PLA-Graphene composite specimens, except in the case of *Y*-axis and Flat orientation. Moreover, PLA-Graphene specimens showed, in overall terms, the best dimensional accuracy in *Z*-axis because of an enhanced interlaminar adhesion, as reported in the previous section.

**Figure 10:**
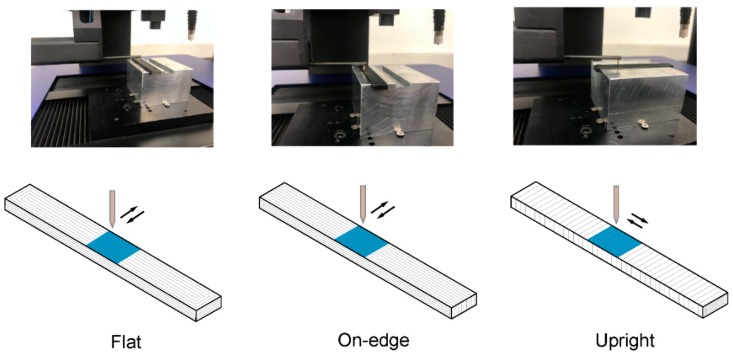
Surfaces texture measurements for the different build orientations.

**Figure 11:**
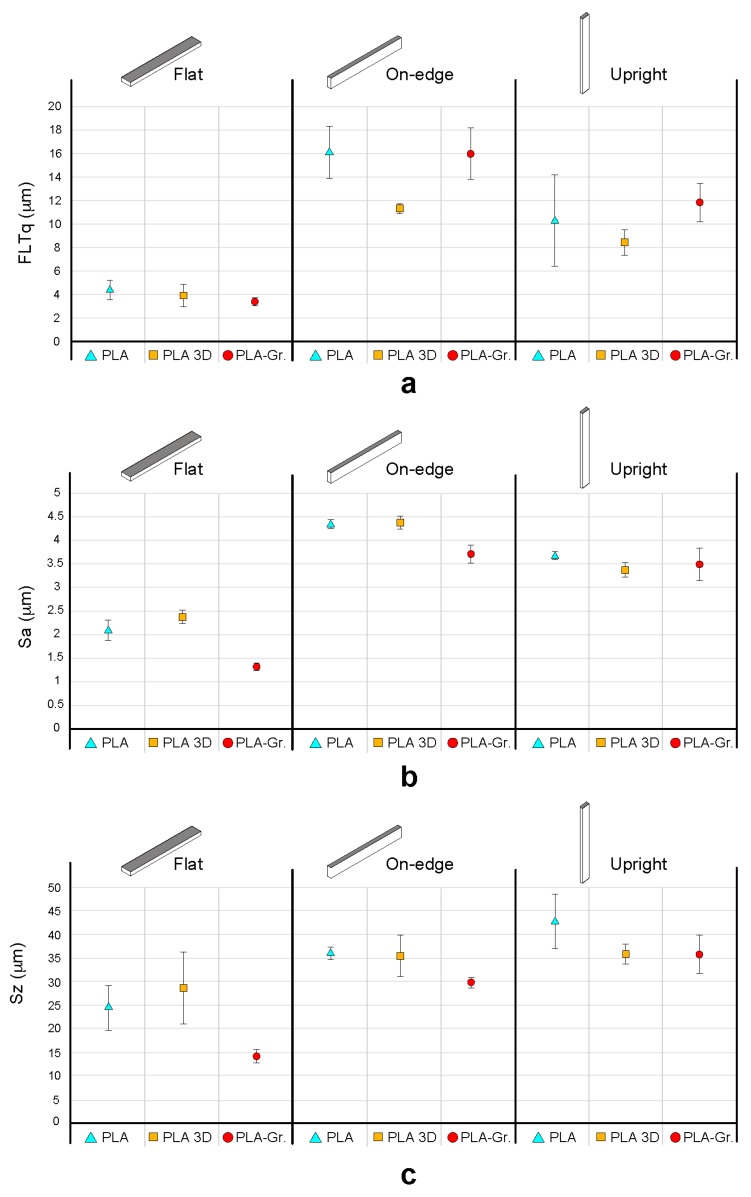
Surface texture results of PLA-based samples as a function of build orientation and the type of material. The results of three samples of each set are shown for comparative purposes: (**a**) Root mean square flatness deviation (Fltq), (**b**) Arithmetic mean height (Sa), (**c**) Maximum height (Sz).

Finally, Figure 10 and Figure 11 show the surface texture analysis for the PLA-based samples. For flatness and surface texture evaluation, a large enough measuring area was required to properly determine the changes in shape and texture. Hence, all specimens were evaluated using the X-Y plane with a sampling area of 10 mm × 10 mm (Figure 10). This configuration enabled to assess different aspects related to build orientation. In the Flat orientation, the flatness and surface texture of a layer involved by X-Y extrusion path and the filament printing process was evaluated. In addition, in On-edge orientation, the layer deposition from Z = 0 mm to Z = 10 mm was evaluated and, finally, in Upright orientation, the layer accumulation from Z = 35 mm to Z = 45 mm, was evaluated.

Table 7 and Figure 11 depict the evolution of flatness deviation (FLTq) and surface roughness (Sa and Sz) for each material and build orientation, showing the average and standard deviation of the three materials and build orientations analyzed in this study. For the flatness deviation (Figure 11a), Flat-oriented samples exhibited much better results than On-edge and Upright orientations, with FLTq values lower than 5 μm in all cases. In Flat orientation, it can be observed similar behavior in the three materials, with FLTq values of 4.32 ± 0.83 μm, 3.82 ± 0.93 μm and 3.35 ± 0.23 μm for PLA, PLA3D and PLA-Graphene, respectively. In the case of On-edge orientation, the lowest flatness deviation was obtained for PLA3D, with FLTq values of 11.24 ± 0.50 μm, whereas PLA and PLA-Graphene exhibited similar FLTq values of 16.08 ± 2.22 and 15.94 ± 2.27 µm, respectively. In the case of Upright orientation, PLA3D showed the best results, with FLTq values of 8.39 ± 1.17 μm compared to 10.23 ± 3.95 μm and 11.74 ± 1.79 μm of PLA y PLA-Graphene, respectively. The surface roughness (Sa) exhibited similar behavior that flatness deviation (Fltq) (Figure 11b), where the best results were obtained for flat orientation, with a significant increase of Sa for On-edge and Upright orientations. This effect was because, in Flat orientation, the surface roughness mainly depended on the printed filament, and in On-edge and Upright orientations depended on the printed layer. In the case of Flat orientation, PLA and PLA3D exhibited similar results, with Sa values of 2.08 ± 0.23 and 2.36 ± 0.14 μm, respectively, while PLA-Graphene showed a significant decrease of roughness, with Sa values of 1.28 ± 0.06 μm. In addition, in the case of On-edge orientation, PLA and PLA3D depicted similar behavior without significant differences, with Sa values of 4.32 ± 0.08 and 4.36 ± 0.14 μm, respectively, and PLA-Graphene showed an improvement, with a Sa reduction of 3.71 ± 0.22 μm. Finally, in the case of Upright orientation, there were not significant differences between the three materials, with Sa values of 3.65 ± 0.05, 3.36 ± 0.15, and 3.48 ± 0.38 μm for PLA, PLA3D and PLA-Graphene, respectively. Finally, Figure 11c shows the analysis of the surface roughness by Sz parameter. It can be shown that the best results were obtained for the Flat orientation. PLA and PLA3D exhibited similar results of Sz values, while PLA-Graphene showed an improved surface roughness in terms of Sz parameter.

In short, PLA-Graphene specimens showed, in overall terms, the best performance in terms of surface texture, particularly when parts were printed in Flat and On-edge orientations.

## 4. Conclusions

The mechanical performance, dimensional accuracy and surface roughness analysis of 3D printed PLA-based and graphene nanoplatelets (GNPs) reinforced composites manufactured by FFF technique have been analyzed. The effect of build orientation and graphene reinforcement were studied in particular. Tensile, three-point bending, Charpy impact and interlaminar shear strength tests were carried out to determine the mechanical response of the 3D printed specimens following ASTM standard recommendations. SEM images of ILSS failure samples were evaluated to determine the effects of GNPs on bonding performance.

It has been shown that the effect of build orientation was of particular significance on the mechanical performance of PLA-based materials. On-edge and flat orientations showed the highest values for maximum tensile and flexural strengths and stiffness, while upright orientation resulted in the lowest ones. There were no significant differences between PLA and PLA3D850 in terms of tensile and flexural behavior. Furthermore, PLA-Graphene composite samples showed the best performance in terms of tensile and flexural stress and stiffness, except in the case of on-edge orientation. GNPs offered higher stiffness with respect to PLA matrix, preventing the shear strain. More specifically, PLA-Graphene composite showed a significant improvement of the tensile behavior over the other two materials in the case of upright orientation. However, the addition of GNPs tended to reduce the impact strength of the PLA composite samples. Finally, PLA-Graphene composite samples showed the highest interlaminar shear strength. These results were in accordance with the enhanced interlaminar adhesion and performance showed by the PLA-Graphene samples under tensile and flexural loading.

Moreover, the addition of graphene nanoplatelets did not affect, in general terms, the dimensional accuracy of the PLA-Graphene composite specimens. They showed, in overall terms, the best dimensional accuracy in *Z*-axis due to enhanced interlaminar performance. Finally, PLA-Graphene specimens showed, in overall terms, the best performance in terms of surface texture, particularly when parts were printed in flat and on-edge orientations.

The results have shown that it is still a challenge to increase the mechanical performance of 3D printed reinforced composite materials with respect to conventional polymer processing methods such as compression or injection molding. A compaction stage after the deposition of the filament would be desirable to reduce porosity. Nevertheless, the properties obtained by 3D printed reinforced composites by FFF are, in general terms, higher than the usual 3D FFF thermoplastics.

In conclusion, using FFF to fabricate 3D printed composites with much higher mechanical performance has become a cutting-edge and interdisciplinary research topic in the last few years. It seems to be a very promising technology with potential for future development. It is a relatively new technique and there is a lack of experimental data on the mechanical performance of structures manufactured by this process, underscoring the need for further research to improve our understanding of the mechanical behavior of 3D printed composites.

## Figures and Tables

**Table 1:** The basic mechanical properties of PLA-based materials used in this work. Standard deviation is depicted in brackets. Typical ranges of mechanical properties for PLA and ABS materials manufactured by FFF technology provided by the manufacturers are also included for comparative purposes [12,13,50].

Mechanical Properties	Smartfil PLA	Smarfil PLA 3D850	PLA-Graphene	PLA	ABS
Tensile strength (MPa)	35.6 (3.8)	53.4 (2.1)	66.8 (1.3)	15.5–72.2	36–71.6
Tensile modulus (MPa)	3420 (62)	3510 (82)	3752 (85)	2020–3550	99.8–2413
Elongation at break (%)	4.2 (0.2)	4.4 (0.3)	2.6 (0.1)	0.5–9.2	3–20
Flexural strength (MPa)	85.2 (2.2)	98.4 (3.1)	98.5 (2.4)	52–115.1	48–110
Flexural Modulus (MPa)	2378 (57)	2404 (42)	2450 (94)	2392–4930	1917–2507
Izod Impact strength (J/m)	29.2 (2.3)	34.6 (3.3)	40.4 (2.9)	27–192	47–174
Density (g/m^3^)	1.24	1.24	1.11	-	-

**Table 2:** Process parameters and their levels used in this work.

Parameters	Value
Layer thickness (mm)	0.12
Feed rate (mm/s)	50
Flow rate (mm^3^/s)	4.8
Top and Bottom thickness (mm)	0.6
Nozzle temperature (°C)	210
Nozzle size (mm)	0.4

**Table 3:** Average tensile and flexural strength and stiffness results of PLA, PLA 3D850 and PLA-Graphene samples for Flat, On-edge and Upright orientation. Standard deviation is depicted in brackets.

Orientation	Material	Tensile Test Results		3-Point Bending Test Results	
		σ_t_ (MPa)	E_t_ (GPa)	σ_f_ (MPa)	E_f_ (GPa)
**Flat**	PLA	49.5 (4.7)	3.543 (0.07)	93.5 (1.4)	2.287 (0.02)
	PLA 3D850	52.9 (1.6)	3.550 (0.01)	95.4 (1.7)	2.221 (0.02)
	PLA-Graphene	63.2 (2.8)	3.597 (0.10)	83.4 (0.5)	2.412 (0.01)
**On-edge**	PLA	66.5 (4.5)	3.430 (0.08)	98.6 (0.5)	2.308 (0.07)
	PLA 3D850	67.3 (1.1)	3.505 (0.06)	99.1 (2.2)	2.362 (0.06)
	PLA-Graphene	61.8 (1.8)	3.590 (0.90)	94.3 (1.5)	2.435 (0.20)
**Upright**	PLA	26.1 (4.3)	3.442 (0.04)	42.3 (5.3)	2.238 (0.02)
	PLA 3D850	27.8 (1.8)	3.532 (0.22)	47.6 (9.4)	2.273 (0.01)
	PLA-Graphene	39.3 (1.1)	3.641 (0.14)	71.1 (8.0)	2.427 (0.04)

**Table 4:** Average Charpy impact strength results of PLA, PLA 3D850 and PLA-Graphene samples for Flat, On-edge and Upright orientation. Standard deviation is depicted in brackets

Orientation	Material	Charpy Impact Strength
		E_C_ (kJ/m^2^)
Flat	PLA	42.5 (2.6)
	PLA 3D850	35.2 (0.9)
	PLA-Graphene	34.3 (1.3)
On-edge	PLA	30.1 (1.9)
	PLA 3D850	27.5 (2.1)
	PLA-Graphene	22.5 (1.5)
Upright	PLA	20.8 (1.4)
	PLA 3D850	20.1 (1.4)
	PLA-Graphene	19.5 (1.2)

**Table 5:** Average ILSS test results of PLA, PLA 3D850 and PLA-Graphene. Standard deviation is depicted in brackets.

Interlaminar Shear Strength τILSS (MPa)	
PLA	14.5 (0.61)
PLA 3D850	14.8 (0.74)
PLA-Graphene	17.1 (1.21)

**Table 6:** Average dimensional accuracy results of PLA, PLA 3D850 and PLA-Graphene samples as a function of build orientation and the type of material. The results of three samples of each set are shown for comparative purposes. Maximum and minimum dimensional deviation of each sample are depicted in brackets.

Orientation	Material	${\bar{D}}_{x}\;(\max,\;\min)$	${\bar{D}}_{y}\;(\max,\;\min)$	${\bar{D}}_{z}\;(\max,\;\min)$
		(μm)	(μm)	(μm)
Flat	PLA	−36 (15, −95)	124 (232, 13)	−103 (−33, −175)
		−103 (−30, −167)	61 (145, −20)	−46 (20, −112)
		−108 (−35, −192)	158 (291, 11)	−112 (−37, −184)
	PLA 3D850	−292 (−218, −375)	16 (132, −92)	22 (118, −93)
		−273 (−200, −337)	29 (104, −35)	51 (127, −33)
		−299 (−234, −360)	−24 (37, −87)	47 (116, −24)
	PLA-Graphene	137 (228, 56)	413 (478, 347)	−36 (50, −108)
		114 (181, 42)	412 (496, 320)	−55 (1, −110)
		166 (238, 101)	397 (484, 308)	−82 (−12, −147)
On-edge	PLA	−188 (−132, −236)	166 (249, 93)	−118 (−47, −191)
		−221 (−148, −295)	186 (295, 89)	−75 (−10, −141)
		−257 (−213, −301)	174 (276, 69)	−108 (−39, −175)
	PLA 3D850	−304 (−243, −364)	251 (326, 172)	−53 (26, −123)
		−288 (−243, −333)	246 (308, 184)	−56 (15, −131)
		−320 (−232, −386)	233 (309, 156)	−95 (−11, −159)
	PLA-Graphene	−253 (−175, −322)	344 (423, 264)	−23 (32, −80)
		−203 (−136, −259)	312 (392, 239)	48 (103, −11)
		−256 (−173, −325)	282 (359, 203)	−3 (51, −42)
Upright	PLA	113 (201, 27)	168 (256, 85)	216 (274, 162)
		23 (87, −43)	70 (176, −51)	170 (218, 119)
		97 (171, 23)	181 (303, 90)	140 (187, 90)
	PLA 3D850	1 (76, −70)	144 (239, 40)	375 (441, 313)
		76 (154, −9)	120 (212, 27)	296 (349, 250)
		−30 (73, −134)	92 (232, −93)	246 (289, 204)
	PLA-Graphene	152 (235, 62)	97 (169, 30)	142 (190, 91)
		21 (103, −60)	57 (119, −3)	135 (198, 80)
		23 (100, −51)	21 (135, −96)	81 (142, 17)

**Table 7:** Average surface texture results of PLA, PLA 3D850 and PLA-Graphene samples as a function of build orientation and the type of materials. Standard deviation is depicted in brackets

Orientation	Material	Fltq	Sa	Sz
		(μm)	(μm)	(μm)
Flat	PLA	4.32 (0.83)	2.08 (0.23)	24.37 (4.99)
	PLA 3D850	3.82 (0.93)	2.36 (0.14)	28.56 (7.80)
	PLA-Graphene	3.36 (0.23)	1.28 (0.07)	14.13 (1.55)
On-edge	PLA	16.08 (2.22)	4.32 (0.08)	35.83 (1.34)
	PLA 3D850	11.25 (0.50)	4.37 (0.14)	35.35 (4.61)
	PLA-Graphene	15.94 (2.28)	3.71 (0.22)	29.70 (1.30)
Upright	PLA	10.23 (3.96)	3.66 (0.05)	42.54 (6.06)
	PLA 3D850	8.39 (1.17)	3.36 (0.15)	35.68 (2.46)
	PLA-Graphene	11.75 (1.79)	3.49 (0.38)	35.69 (4.23)
